# Vortex Analysis of Intra-Aneurismal Flow in Cerebral Aneurysms

**DOI:** 10.1155/2016/7406215

**Published:** 2016-11-07

**Authors:** Kevin Sunderland, Christopher Haferman, Gouthami Chintalapani, Jingfeng Jiang

**Affiliations:** ^1^Department of Biomedical Engineering, Michigan Technological University, Houghton, MI, USA; ^2^Siemens Medical Solution USA, Inc., Heffernan Estate, IL, USA

## Abstract

This study aims to develop an alternative vortex analysis method by measuring structure ofIntracranial aneurysm (IA) flow vortexes across the cardiac cycle, to quantify temporal stability of aneurismal flow. Hemodynamics were modeled in “patient-specific” geometries, using computational fluid dynamics (CFD) simulations. Modified versions of known *λ*_2_ and *Q*-criterion methods identified vortex regions; then regions were segmented out using the classical marching cube algorithm. Temporal stability was measured by the degree of vortex overlap (DVO) at each step of a cardiac cycle against a cycle-averaged vortex and by the change in number of cores over the cycle. No statistical differences exist in DVO or number of vortex cores between 5 terminal IAs and 5 sidewall IAs. No strong correlation exists between vortex core characteristics and geometric or hemodynamic characteristics of IAs. Statistical independence suggests this proposed method may provide novel IA information. However, threshold values used to determine the vortex core regions and resolution of velocity data influenced analysis outcomes and have to be addressed in future studies. In conclusions, preliminary results show that the proposed methodology may help give novel insight toward aneurismal flow characteristic and help in future risk assessment given more developments.

## 1. Introduction

A weak or thin spot on cerebral arteries, resulting in the bulging or ballooning (dilation) of that vessel, is known as an intracranial aneurysm (IA). Most common forms of IAs are saccular and fusiform IAs. These irregular dilations act like cavities around the blood stream and, therefore, induce flow disturbance and subsequent destructive vascular remodeling [1] in and around IAs. IAs are a potentially life-threatening vascular malformation affecting an estimated 3% of the population [2]. According to the US National Institute of Neurological Disorders and Stroke, the incidence of reported IA rupture is about 0.001%. In other words, there are approximately 30,000 people who could suffer a stroke from ruptured cerebral aneurysms each year in the United States alone. The severe consequences of aneurysm rupture, the cost and risk of aneurysm treatment, the low statistical risk of aneurysm rupture (1% or less per year), and the lack of reliable parameters predictive of the risk of aneurysm rupture, all motivate the continued search for a method to differentiate those aneurysms which are likely to rupture from those at a low risk of rupture.

Currently, geometrical and morphological parameters of IAs (e.g., size and irregular shape) have been used as the basis for clinical predictions of aneurysm rupture, subsequently directing the course of clinical intervention(s), according to recommendations by multiple healthcare professional organizations [3–5]. However, those predictions are not highly accurate. For instance, based on receiver operating characteristic analysis, the size ratio and aneurysm angle had an area under the curve values of 0.83 and 0.85, respectively [6]. Recently, attention has been devoted to aneurysmal hemodynamics, since it is well known that the origin and natural history of IAs are closely associated with disturbed hemodynamics [7, 8]. Consequently, phase-contrast magnetic resonance imaging (PC-MRI) [9, 10] has been used to measure aneurysmal flow and examine factors relevant to the development and progression of IAs* in vivo*. But disturbed flow in and around IAs makes this task difficult. Recall that individual protons in complex and disturbed aneurysmal flow have incoherent velocities (at the subgrid level) and those components cannot be resolved by one “averaged” velocity measurement from a relatively large resolution cell (*at 1 mm scale*). Therefore, such measurement errors and potential artifacts can adversely affect the accuracy of PC-MRI results. In parallel to research efforts of PC-MRI flow imaging in and around IAs, blood flow parameters simulated from “patient-specific” computational fluid dynamics (CFD) simulations [11] have also drawn a lot of interest by the clinical and research community [12, 13]. Hemodynamic factors such as wall shear stress [14], oscillatory shear index [15], flow impingement [13], and flow stability [16] have emerged as potential parameters correlating to the risk of IA rupture. Among those efforts, Byrne et al. [16] found that aneurysmal flow, classified by its spatial complexity and temporal stability using vortex core lines, is closely correlated with the risk of aneurysm rupture. Particularly, they concluded that “ruptured aneurysms had more complex and more unstable flow patterns than unruptured aneurysms.” In their work, Byrne et al. have used proper orthogonal decomposition [17] of time-resolved velocities to characterize temporal flow stability. In another study, Kohler et al. [18] presented another approach to visualizing vortex flow using line predicates. However, their technique was primarily for visualization of swirling flow in relatively large arteries such as the aorta and pulmonary artery.

In this study, we intended to develop an alternate technique, also based on the flow vortex core analysis, to characterize the presence, destruction, and motion of spatial swirl flow patterns during the cardiac cycle. More specifically, we implemented an image processing algorithm to first delineate the vortex core region(s) and then quantitatively investigate temporal flow stability by tracking segmented vortex cores over the cardiac cycle. The vortex extraction method was based on velocity data on a rectilinear grid; potentially, this extraction method could be used for PC-MRI measured velocity fields, as well. Toward this end, the primary purpose of this work is to explore and analyze the structural characteristics of flow vortexes as a possible means to complement current assessment methods of temporal flow stability.

The rest of the paper is structured as follows. In Materials and Methods, using original 3D velocity estimates, the proposed image processing method is described in detail. To demonstrate its feasibility, the proposed method is applied to “patient-specific” CFD simulated velocity data using two distinct solvers (a commercial CFD solver using unstructured meshes and a research prototype solver using the Lattice-Boltzmann Method [LBM]). As part of this preliminary study, two CFD solvers were selected in order to investigate whether or not results of the proposed method of vortex core analysis may be influenced by CFD solvers. Outcomes of this feasibility study are reported in Results, followed by Discussions and Conclusions.

## 2. Materials and Methods

**Figure 1:**
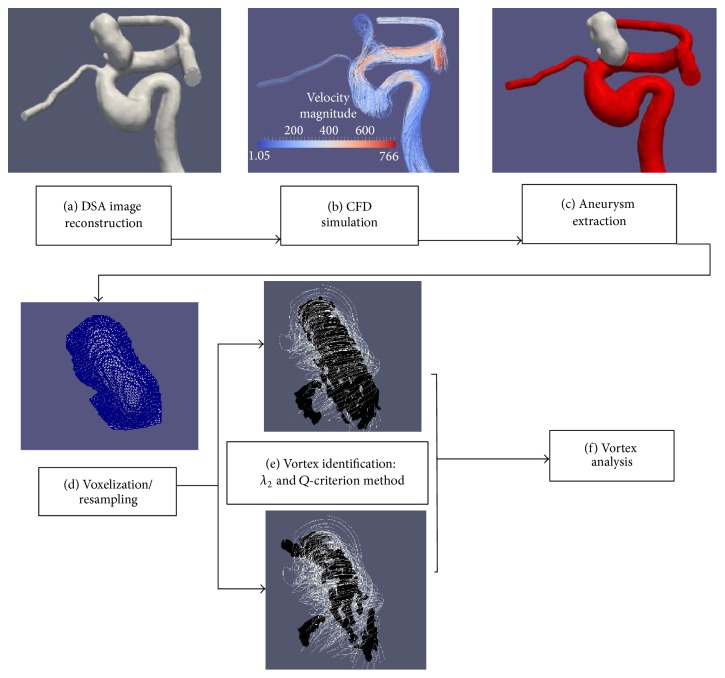
Main steps in the generation and analysis of vortex core structures: (a) arterial geometry generated and segmented from a DSA scan; (b) volumetric mesh generation and CDF simulation on the segmented structure; (c) manual extraction of an aneurysm surface from the artery (gray denotes the area of an aneurysm and red denotes the parent artery); (d) extracted aneurysm surface masked over the simulated data to isolate aneurysm flow velocity and resampled at various voxel sizes (0.1–0.8 mm); (e) the *λ*_2_ and *Q*-criterion methods identify vortexes for 21 equally spaced data points over the cardiac cycle; (f) analysis of vortex: characteristics and DOV (stability) between each cardiac step's vortex core and the cycle-averaged vortex core. In (e), identified vortex core regions are segmented out using the classic marching cube algorithm. In (e), while the white color denotes streamlines of the aneurysmal flow, the black color is used to indicate vortex structures using the two above-mentioned methods—*λ*_2_ and *Q*-criterion methods.

Extraction and tracking of vortex cores are built around time-resolved velocity fields obtained from “patient-specific” CFD simulations.  Figure 1 shows two major steps: (1) hemodynamics simulations and (2) vortex core extraction and analysis. The hemodynamic flow was first simulated in vascular models reconstructed from 3D-Digital Subtraction Angiography (DSA) scans and then those time-resolved 3D velocity fields were used to determine vortex core regions within the aneurysmal sac. Of note, the identification of vortex core regions was derived from two known methods: *λ*_2_ method [19] and the *Q*-criterion method [20]. All identified vortex core regions were then segmented out using the classic marching cube algorithm [21] to form 3D triangulated surfaces. Degree of vortex overlap (DVO) among the segmented vortex cores over the cardiac cycle and variations of the number of vortex core regions over the cardiac cycle were used to assess destruction and motion of spatial swirl flow patterns within the aneurismal sac over the cardiac cycle.

### 2.1. Modeling of “Patient-Specific” Hemodynamics

#### 2.1.1. Image Segmentation

**Figure 2:**
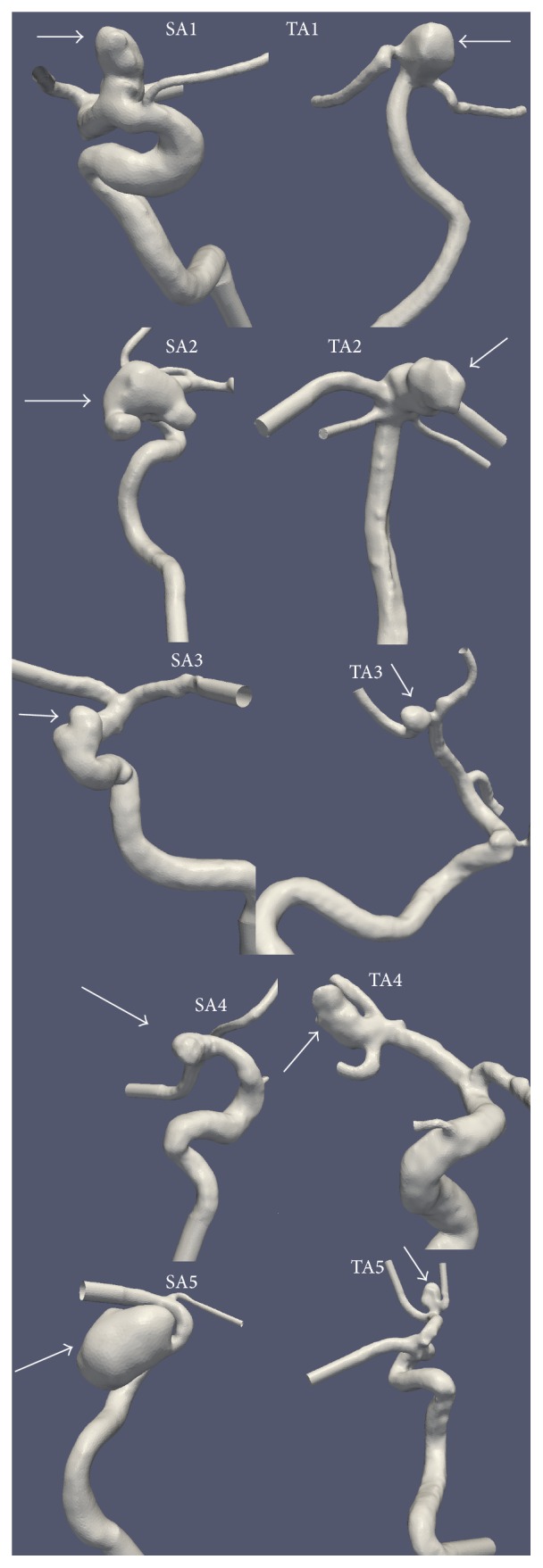
Five sidewall aneurysms (SAs) and five terminal aneurysms (TAs) used in this study. All geometries were reconstructed from high-resolution 3D-Digital Subtraction Angiography (DSA). Arrows point to IAs.

As shown in Figure 2, 10 cases of IAs were arbitrarily selected from our internal database: in five cases, each has one single terminal aneurysm (TA); in the other five cases, each contains single sidewall aneurysm (SA). Initial 3D-DSA data files were loaded into a commercially available image segmentation package (Mimics Innovation Suite, version 17, Materialise Inc., Leuven, Belgium) where intensity-based image segmentation was first used to isolate the regions of interest and create a volumetric surface of patients' vascular structures. During this process, a first-order Laplacian smoothing filter in the 3-Matic Software (version 9, Materialise Inc., Leuven, Belgium) was used to reduce irregularities caused by imaging artifacts, while preserving surface shape. Then, manual editing (e.g., removal of kissing vessels) and visual inspection were performed to ensure the integrity of all vessel geometries if necessary. Downstream vessels were shortened in order to reduce computing time. The longest possible upstream section, proximal to an aneurysm, was left intact to help reduce the effects of the inlet(s) and plug-flow alterations [22]. Cylindrical flow extensions (at least 6 times of the vessel diameter) were added to each model (both inlets and outlets) to mitigate influences of boundary conditions using the open-source Vascular Modeling Toolkit (VMTK) software (version 1.2) [23].

#### 2.1.2. Mesh Generation

Each processed vascular surface model (see Figure 2) was converted into an unstructured, 3D, tetrahedralized volumetric mesh using an open-source mesh generator, Tetgen (version 1.4.2) [24]. The mesh generation process was done by an in-house Python script derived from the VMTK program (version 1.2) [23]. Approximately, 1 million computing cells were used per case, with the average mesh size as 0.0022 mm^3^. The typical edge length of resultant tetrahedrons was roughly 0.12 mm. Mesh sensitivity tests were performed to ensure that results were not sensitive to the mesh size selected.

#### 2.1.3. CFD Simulations

To compute velocity waveforms in and around an aneurysm using the ANSYS-FLUENT solver (version 14.0; ANSYS-FLUENT Inc., Lebanon, NH), we solved the time-dependent incompressible, 3D Navier-Stokes equations [i.e., (1)] for the 3D meshed vessel geometry. The equations for velocity are written as

(1) ∇→·u→=0,ρ∂u→∂t+ρu→·∇→u→=−∇→p+μ∇2u→,

where $\vec{u}$ is the three-dimensional velocity vector, *ρ* is the blood density, *p* is the pressure, and *μ* is the viscosity. In the ANSYS-FLUENT CFD solver, the pressure-velocity coupling in (1) is obtained using the SIMPLEC algorithm [25]. The explicit time-marching second-order scheme with a time step of 1 × 10^−3^ seconds (approximately 1000 steps per cardiac cycle) was used for the computations. Although this time step is still relatively coarse, it seems adequate to capture gross flow features. The tetrahedralized volumetric meshes described above were used by the ANSYS-FLUENT solver. More details can be found in our previous publications [26, 27].

The Siemens research prototype CFD solver (version 4.0, Prototype: not for diagnostic use, Siemens Medical Solution, Inc., IL) is based on the Lattice-Boltzmann Method (LBM) with a Multiple Relaxation Time (MRT-LBM) approximation [28]. More details can be found in a previously published paper [28]. In the current implementation, a graphic processing unit (GPU) was used to accelerate the flow simulations. Prior to CFD simulations using the Siemens solver, IA models defined by water-tight 3D surface triangles were automatically discretized with cubical voxels; a smooth level-set function was created to differentiate the vessel and nonvessel regions for each IA model. For each IA, its complex geometry was represented by the zero-crossing of the above-mentioned smooth level-set function, which was used for the accurate imposition of the boundary conditions. All subsequent LBM computations were performed on a Cartesian grid, where the global resolution and time step size were automatically adapted by the Siemens LBM solver for ensuring computational stability. Of note, the exact same vessel geometries (STL files) were used to generate volumetric meshes (for the ANSYS-FLUENT solver) and voxel discretization (for the LBM solver). It is important to note that the Siemens research CFD solver is only an investigative prototype and not commercially available.

In both solvers, vessel walls were assumed rigid with a no-slip boundary condition, and blood flow was considered incompressible and Newtonian. The dynamic viscosity was 0.004 kg/m-s and the mass density of blood was 1050 kg/m^3^. The inlet boundaries of all models were located in the internal carotid artery or the basilar artery. The zero-pressure condition was used for all outlets. Two pulsatile flow rate waveforms, at a heart rate of 60 bpm, derived from magnetic resonance measurements and taken from Gwilliam et al. [29] were used since patient-specific waveforms were not available. Of note, one waveform was adopted to represent the pulsatile flow at the BA (TA1 and TA2; see Figure 2) and the other waveform was selected to represent the pulsatile flow at the ICA (all other cases; see Figure 2). Each case had one velocity waveform scaled according to the vessel's inlet cross-sectional area, standardizing the mean volumetric flow rate of 280 mL/min (ICA) or 180 mL/min (BA). Our choice of mean flow rates is consistent with physiological flow rates available in the MR literature [30, 31]. Four cardiac cycles were simulated per case as a means to reduce initial transient conditions, and only the final cycle was saved. Twenty-one equally spaced data points across the final cardiac cycle were saved for subsequent data analysis detailed below.

### 2.2. Vortex Core Extraction and Analysis

All computational methods for extraction of vortex core regions and analysis of those vortex cores were performed using in-house scripts (C++ and Python) that were derived from the open-source VTK/VMTK (version 1.2) software package [23]. Statistical and correlation analysis was performed using MATLAB scripts.

#### 2.2.1. Aneurysm Extraction and Voxelization of Aneurismal Velocity Data

A published method [32] was used to semiautomatically isolate and extract the aneurysm sac (see white colored surface in Figure 1(c)). The isolated aneurysm sac was sealed at the orifice and converted to a binary mask that is spatially registered with volumetric velocity data. The mask allowed us to analyze intra-aneurysmal velocity data only. To verify the intrarater reliability of proper sectioning of aneurysm masks, two separate users (Kevin Sunderland and Christopher Haferman) sectioned all aneurysms, and Bland-Altman plots [33] were performed on the mask volumes and ostium areas to determine the similarity between chosen masks. Once no significant difference was ensured between sectioned masks, one user was chosen at random and all masks from that user were implemented in the rest of the study.

Simulated ANSYS-FLUENT velocity data are typically located on an unstructured grid. Therefore, resampling of the simulated velocity data was transformed onto a (3D) rectilinear grid, while the velocity data obtained from the Siemens research CFD solver was already on a rectilinear grid. Both data sets were resampled onto a rectilinear grid of 0.2 mm voxel size using the vtkProbeFilter function provided by the open-source VTK package (Kitware Inc., NY, USA), as a means to standardize the resolution of each data set.

In addition, two cases of simulated velocity data using ANSYS-FLUENT were resampled at varying voxel sizes (0.1–0.8 mm) to determine how spatial resolution of velocity data may impact the characteristic structure of generated vortex cores and stability over the cardiac cycle. Determining the changes that voxel size could have on vortex structure characteristics would help to establish proper analysis parameters for this technique.

#### 2.2.2. Vortex Identification

Multiple algorithms exist to locate and extract vortexes in the CFD literature [34]. This study employed two classic methods: *λ*_2_ method developed by Jeong and Hussain [19] and the *Q*-criterion by Hunt et al. [20]. More specifically, given a CFD-simulation velocity field $\vec{u}\left(x,t\right)$, the velocity gradient can be decomposed as follows [35]:

(2) $$\begin{array}{c} \nabla \vec{u}=S+\Omega , \end{array}$$

where $S=\left(1/2\right)\left[\left(\nabla \vec{u}\right)+{\left(\nabla \vec{u}\right)}^{T}\right]$ is the rate of strain tensor and $\Omega =\left[\left(1/2\right)\left(\nabla \vec{u}\right)-{\left(\nabla \vec{u}\right)}^{T}\right]$ is the vorticity tensor.

Hunt et al. [20] defined a vortex as a spatial region where

(3) $$\begin{array}{c} Q=\frac{1}{2}\left[\;{\left|\;\Omega \;\right|}^{2}-{\left|\;S\;\right|}^{2}\;\right]>0. \end{array}$$

The vortex core defined by Hunt et al. (i.e., (3)) essentially denotes locations where the Euclidean norm of the vorticity tensor dominates.

Jeong and Hussain suggested that the vortexes are regions where

(4) $$\begin{array}{c} \lambda_{2}\left(\;S^{2}+\Omega^{2}\;\right)<0. \end{array}$$

In (4), *λ*_2_(*A*) means the second intermediate eigenvalue of the 3 × 3 tensor *A*. The tensor is symmetric, and therefore all three eigenvalues are real.

In this study, we attempted to use [normalized] $\overset{̿}{Q}$ and $\overset{̿}{\lambda_{2}}$ values to condense the distributions of original *Q* and *λ*_2_ values so that the subsequent extraction of vortex cores could be reliably performed. Mathematically, this normalization process can be written as follows:

(5) Qx,t̿=Qx,tu→x,t2,λ2x,t̿=λ2x,tu→x,t2,

where $\left|\vec{u}\left(x,t\right)\right|$ is the amplitude velocity value.

Once all *Q* and *λ*_2_ values, including [normalized] $\overset{̿}{Q}$ and $\overset{̿}{\lambda_{2}}$ values, were determined within the dome of the aneurysm, the classic marching cube algorithm [36] was used to extract the vortex core regions and convert them to surface meshes (see Figure 1(e)). In order to extract a vortex core region, the mean value of interest (*Q* [positive] or *λ*_2_ [negative]) for each case was used as a threshold value.

To determine the ideal vortex identification methodology and desired range of analysis parameters, each methodology (standard *λ*_2_, [normalized] $\overset{̿}{\lambda_{2}}$, *Q*-criterion, and [normalized] $\overset{̿}{Q}$-criterion) was run with 5 different threshold values and their resultant vortex structure was analyzed. The 5 threshold values were selected from the following respective ranges: (1) [0.1, maximum] for *Q*-criterion and [normalized] $\overset{̿}{Q}$-criterion and (2) [minimum, −0.1] for standard *λ*_2_ and [normalized] $\overset{̿}{\lambda_{2}}$ methods in each case. In other words, in an ascending order, selected five (5) threshold values were [mean − STD, mean − (STD/2), mean, mean/2, and mean/4] for the standard *λ*_2_ and [normalized] $\overset{̿}{\lambda_{2}}$ methods and [mean/4, mean/2, mean, mean + (STD/2), and mean + STD] for the *Q*-criterion and [normalized] $\overset{̿}{Q}$-criterion methods.

In order to reduce the appearance of small, isolated areas being mistaken for the dominant vortex structure, only extracted vortex core regions which have a greater volume than 0.5 mm^3^ were counted.

### 2.3. Data Analysis

#### 2.3.1. Vortex Stability over the Cardiac Cycle

**Figure 3:**
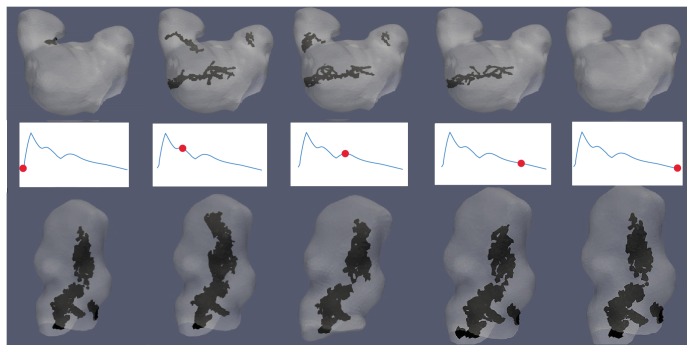
Example of variations to vortex core structure across 5 data points along the cardiac cycle: marked waveforms represent a point in the cardiac cycle for each data point. The black structure is the extracted vortex core(s) while gray is an aneurysm. The top row is for SA2 which had a lower DVO (less stable) than the bottom row from TA2 which had a higher DVO (more stable). All cores were extracted using the [normalized] $\overset{̿}{Q}$-criterion method. The mean [normalized] $\overset{̿}{Q}$ threshold values were used to extract the vortex core and only vortex cores with a volume ≥ 0.5 mm^3^ were saved.

For each CFD simulation, the vortex structure was generated for all 21 data points along the cardiac cycle, as well as a structure based on the cycle-averaged vorticity. The DVO between each data point's vortex structure and the cycle-averaged vortex structure were calculated. The mean and standard deviation (STD) of the DVO across all data points for the cardiac cycle were calculated to quantify the average temporal stability of the vortex structure. “Stability” is referring to the tendency of a vortex structure to shift or change over the course of a cardiac cycle. A visual representation of variations in vortex structure (temporal and spatial) over a cardiac cycle can be seen in Figure 3.

#### 2.3.2. Geometric and Hemodynamic Parameters of IAs

To discern the independence of this method in comparison to established methods for aneurysm analysis, it was explored whether these findings correlated with a number of geometrical [6] and hemodynamic [37] characteristics used to predict IA rupture. Six geometrical characteristics and two hemodynamic characteristics were calculated for each IA using in-house VMTK scripts.

*Geometric Characteristics*

1. Aneurysm volume (mm^3^)
2. Ostium (neck) area (mm^2^): surface area of the opening of an aneurysm
3. Ostium circumference (mm)
4. Aneurysm height (mm): midpoint of ostium to furthest point on aneurysm wall
5. Aspect ratio: aneurysm height/ostium diameter (widest distance across ostium)
6. Aneurysm volume/ostium area (mm) [38]

*Hemodynamic Characteristics*

1. Spatially Averaged Oscillatory Shear Index (SA-OSI)
2. Spatially and Temporally Averaged Wall Shear Stress (STA-WSS)

The calculated geometric values were verified by performing a secondary measurement on the removed aneurysm surface in the 3-Matic Software package. The geometric parameters were collected for all aneurysm masks that were originally segmented from two users (Kevin Sunderland and Christopher Haferman). Once no significant differences were detected between those two users' masks, their averaged geometrical values were used for this study.

Wall shear stresses [WSSs] obtained from the ANSYS-FLUENT solver were first collected from each aneurysm wall for each data point along the cardiac cycle. Then, the WSSs of each spatial location on the aneurysm wall, for each data point along the cardiac cycle, were temporally averaged to obtain the time-averaged wall shear stress for the spatial location. Spatial averaging of all TA-WSSs on the aneurysm wall was performed to obtain the STA-WSS for each aneurysm. To measure the WSS directional oscillations over a cardiac cycle, the quantitative metric known as oscillatory shear index (OSI) was calculated using the following equation [37]:

(6) $$\begin{array}{c} OSI=\frac{1}{2}\left(\;1-\frac{\left|\int_{0}^{T}\tau_{w}dt\right|}{\int_{0}^{T}\left|\tau_{w}\right|dt}\;\right), \end{array}$$

where *T* is the number of data points along the cardiac cycle and *τ*_*w*_ is the instantaneous wall shear stress vector. Spatial averaging of OSI (i.e., SA-OSI) for each aneurysm was calculated.

Pearson's linear correlation was performed to compare the mean DVO and change of the number of vortex cores over the entire cardiac cycle to the aforementioned geometrical and hemodynamic parameters. Only the cases sampled at a resolution of 0.2 mm (voxel) were used for this analysis.

## 3. Results

Results presented in Sections 3.1–3.4 were based on the ANSYS-FLUENT solver. In Section 3.5, the ANSYS-FLUENT results were quantitatively compared to those obtained from the Siemens LBM research CFD solver.

### 3.1. Comparison of Analysis Methods

**Figure 4:**
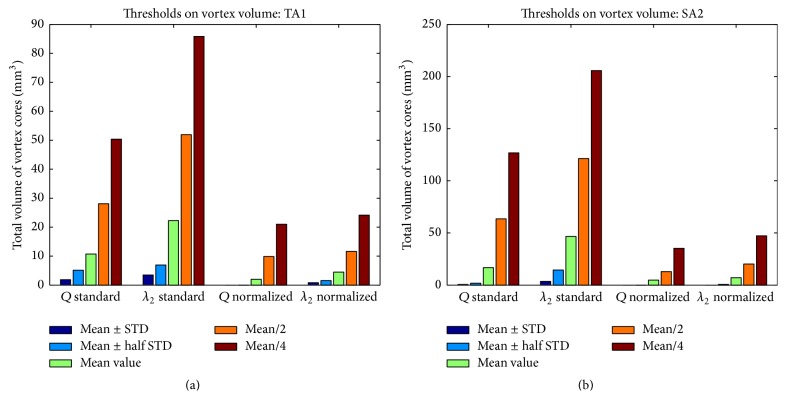
Plots representing variations of identified vortex volumes due to the selection of 5 different threshold values in (a) TA1 and (b) SA1. Threshold values were tested for four vortex extraction methods: standard *Q*-criterion, standard *λ*_2_ method, [normalized] $\overset{̿}{Q}$-criterion, and [normalized] $\overset{̿}{\lambda_{2}}$ method. Geometries of SA2 and TA1 are displayed in Figure 2. Selected threshold values were mean/4, mean/2, mean, mean ± (STD/2), and mean ± STD (positive sign for *Q*-criterion and [normalized] $\overset{̿}{Q}$-criterion methods and negative sign for *λ*_2_ and [normalized] $\overset{̿}{\lambda_{2}}$ methods).

**Figure 5:**
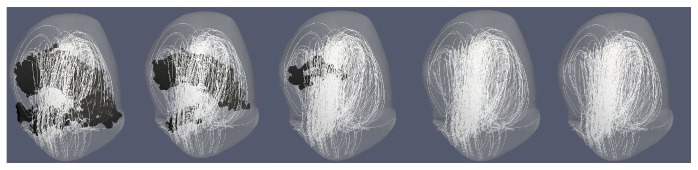
Plots illustrating the impact on extracted vortex cores (i.e., the black surface in each plot) due to changes of isosurface threshold values. From left to right: mean/4, mean/2, mean, mean + (std/2), and mean + std. All images were from TA case 1, [normalized] $\overset{̿}{Q}$ with a voxel resolution of 0.2 mm. At increased threshold values (≥mean + std/2) a reduction of identified vortex core structures occurred, sometimes not identifying any areas of vortex. All images came from the [normalized] $\overset{̿}{Q}$-criterion methodology, case TA1 (see Figure 2), and cardiac cycle-averaged vorticity data.

As shown in Figure 4, choosing high *Q* (or [normalized] $\overset{̿}{Q}$) values or low *λ*_2_ (or [normalized] $\overset{̿}{\lambda_{2}}$) values significantly reduced the sizes of identified vortex core regions. Across all 10 cases studied, setting the threshold larger than mean ± 0.5*∗*STD resulted in a breakdown in the overall identified vortex volume, with no areas of vortex being identified in some cases using the normalized methods (see Figure 4(b)). Extending the threshold past mean/2 (increasing the range of values) resulted in a significant increase in identified vortex volume, as regions corresponding to weaker swirling flow were included as part of the vortex core. A visual representation of these changes can be seen in Figure 5.

It is worth noting that, in terms of the absolute volume change, the [normalized] $\overset{̿}{Q}$-criterion and [normalized] $\overset{̿}{\lambda_{2}}$ method provided more consistent results (i.e., smaller changes due to the threshold variation), as shown in Figures 4(a) and 4(b). This is because the standard *Q*-criterion and standard *λ*_2_ methods resulted in a broader area of swirling flow identified as vortex core in relation to their normalized counterparts.

**Figure 6:**
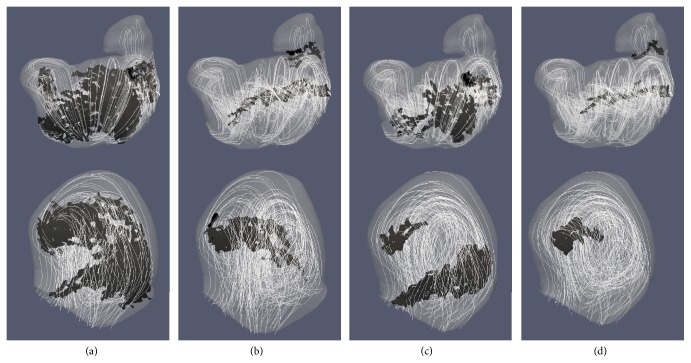
Comparison of identified vortex cores from the four mentioned methods: (a) standard *λ*_2_, (b) [normalized] $\overset{̿}{\lambda_{2}}$, (c) standard *Q*-criterion, and (d) [normalized] $\overset{̿}{Q}$-criterion. Top row is from SA2 case, and bottom row is from the TA1 case. Velocity streamlines were added to represent simulated flow patterns. Geometries of SA2 and TA1 can be found in Figure 2. Threshold values for each case were the mean (for their representative value), and only extracted vortex cores with a volume > 0.5 mm^3^ are shown. Figures are from each method's vorticity averaged data.

Comparison of the extracted vortexes with velocity streamlines in TA1 and SA2 showed that the normalized methods better identified the central region of swirling flow as part of the vortex without including large portions of the weaker, outer swirling flow, as shown in Figure 6. For subsequent data analysis, the [normalized] $\overset{̿}{Q}$-criterion method was chosen using each case's mean [normalized] $\overset{̿}{Q}$ value as the threshold value to identify the vortex core while excluding weaker flow patterns from analysis.

### 3.2. Voxel Size on Vortex Characteristics

**Figure 7:**
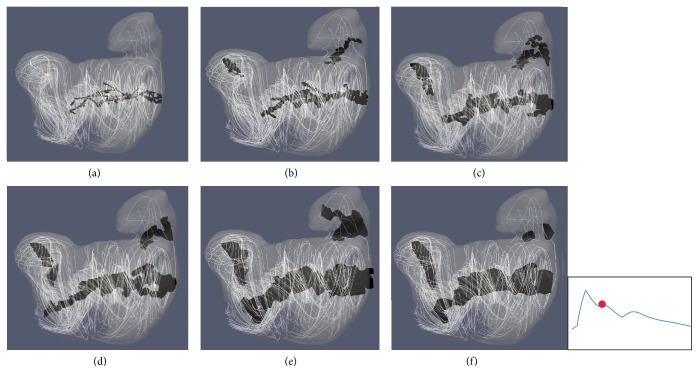
Visual comparison of the impact of voxel size on extracted vortex core structures for case SA2. Voxel sizes: (a) 0.1 mm, (b) 0.2 mm, (c) 0.3 mm, (d) 0.4 mm, (e) 0.6 mm, and (f) 0.8 mm. All structures were extracted using the [normalized] $\overset{̿}{Q}$-criterion method, a threshold value of the mean [normalized] $\overset{̿}{Q}$ value per case, and only cores with a volume > 0.5 mm^3^ were saved. The marked waveform shows the data point in the cardiac cycle used for extracting the structures.

**Figure 8:**
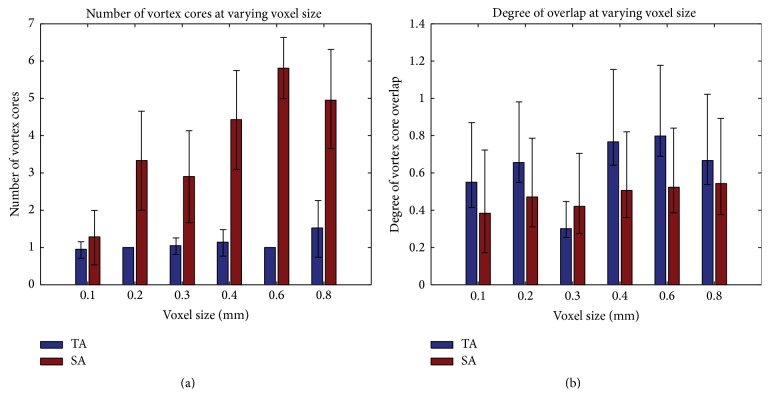
Alterations to vortex core characteristics over different voxel sizes ranged from 0.1 mm to 0.8 mm: (a) changes to the number of vortex cores and (b) changes to DVO. Error bars stand for ±one STD of respective measurements over a cardiac cycle.

Two aneurysm cases studied in the previous subsection (i.e., SA2 and TA1) were resampled at varying voxel sizes and analyzed with the [normalized] $\overset{̿}{Q}$-criterion method to determine how spatial resolution of velocity data impacts vortex core analysis. In order to reduce the appearance of small, isolated areas being mistaken for the dominant vortex structure, only connected vortex core regions which have a greater volume than 0.5 mm^3^ were counted. Through a visual inspection, we found that as voxel size changed, structural characteristics of the vortex core(s) were altered, which could lead to a misinterpretation of the temporal flow stability within an aneurysm. As shown in Figure 7, a high resolution (<0.2 mm) causes the vortex structure to become more fragmented, which may subsequently cause areas of vortex core to be overlooked as their total structural volume may now be smaller than 0.5 mm^3^. By contrast, in lower resolutions (≥0.6 mm), the overall broad structure of extracted core(s) may dwarf subtle changes that could occur in flow patterns leading to a seemingly more stable vortex structure.  Figure 8 shows how changes to voxel sizes impact the mean DVO and the mean number of cores over the cardiac cycle.

For the purposes of a concise presentation of the rest of study, only the data with a resolution of 0.2 mm were analyzed in an attempt to analyze the finer details of the hemodynamic flow patterns, all while still identifying the dominant areas of vorticity.

### 3.3. Vortex Core Parameters in 10 IA Cases

Table 1 shows a summary of the numbers of vortex cores and DVO values among 10 IA cases investigated. Overall, the mean DVO values were similar in TAs (0.50 ± 0.15), as compared to those in SAs (0.41 ± 0.15) with no statistical difference (*p* = 0.37 using a *t*-test) between aneurysm types. The mean numbers of vortex cores seemed slightly higher in SAs (2.35 ± 1.89) as compared to those in TAs (1.88 ± 1.26) but lacked statistical significance (*p* = 0.68 using a *t*-test).

### 3.4. Vortex Correlation with (Aneurysm) Geometrical Parameters

Pearson's linear correlation was used to determine the connection between vortex characteristics (number of vortex cores and DVO) and aneurysm geometric parameters.  Table 2 shows that there are high positive correlations between the number of vortex cores within the aneurysm sac and the volume (*ρ* = 0.995, *p* ≤ 0.001), height (*ρ* = 0.906, *p* = 0.0334), and volume to ostium ratio (*ρ* = 0.998, *p* < 0.001) of SA, whereas no statistically significant correlation exists in TAs between the number of cores and any geometrical parameters. In contrast, no statistically significant correlation exists in SAs or TAs between the DVO values and any geometrical parameters.

To account for the possibility (or lack thereof) of correlations between vortex characteristics and geometrical parameters being attributed to the low number of cases for TA and SA, the data was combined and correlations were recalculated. When data from both aneurysm types were combined, correlations were found between the mean number of cores in relation to 3 geometrical parameters: volume (*ρ* = 0.81, *p* = 0.0042), height (*ρ* = 0.83, *p* = 0.0029), and volume/ostium ratio (*ρ* = 0.83, *p* = 0.0032). Slight negative correlations were also found between the mean DVO of the combined data and aneurysm ostium area (*ρ* = −0.63, *p* = 0.049) and the ostium circumference (*ρ* = −0.64, *p* = 0.046).

**Figure 9:**
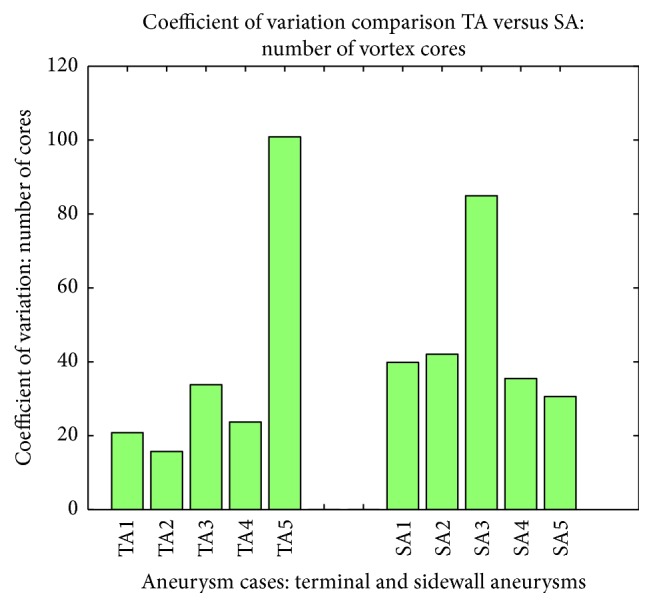
Relative variation for the change in the number of cores over the cardiac cycle or all 10 IA cases.

Due to the significant volume difference between individual aneurysms (and number of vortex cores), the coefficient of variation (STD/mean)*∗*100 was calculated for each case to normalize their relative change of cores over the cardiac cycle (Figure 9). The large spike of the above-mentioned variation in SA3 and TA5 is due to the fact that no coherent cores (>0.5 mm^3^) were detected for a number of data points during the cardiac cycle, thereby causing a reduced mean and increased standard deviation, resulting in a coefficient of variation near or above 100. As shown in Table 3 there only exists a correlation between SAs and their aspect ratio (*ρ* = −0.94, *p* = 0.016) when dealing with the coefficient of variations (vortex cores). All other geometrical parameters held no significant correlation with stability in number of cores across the cardiac cycle. The lack of significant correlations indicates that using the aneurysm geometric parameters (outside of possibly using aspect ratio when dealing with SA) to infer the construction or destruction of vortex cores over the cardiac cycle may not be an ideal methodology.

### 3.5. Comparison with Hemodynamic Parameters

Pearson's linear correlation was also performed to compare the vortex core characteristics to the hemodynamic properties of STA-WSS and SA-OSI present in the aneurysms for this study. Upon initial analysis, no statistically significant correlation was seen in either aneurysm type when comparing the mean OSI across a whole aneurysm, to both the coefficient of variation (number of cores) and the mean DVO. When comparing the STA-WSS values, a significant correlation was only seen with the coefficient of variation for SAs (*ρ* = 0.90, *p* = 0.04). Yet when all data from both IAs were combined, no statistically significant correlative values could be seen for WSS when dealing with coefficient of variations (cores) or the mean DVO across the cardiac cycle. Calculated values for all correlations can be seen in Table 4.

### 3.6. Comparison of Two Different CFD Solvers

**Figure 10:**
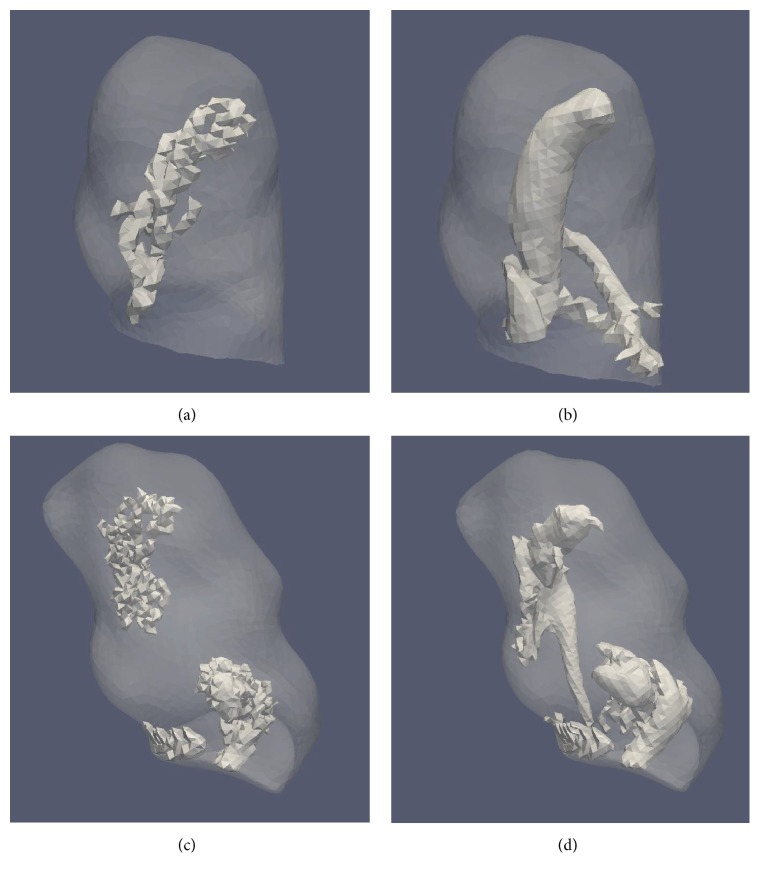
Vortex cores identified from two (SA4 and TA2) ANSYS-FLUENT-simulated cases (a and c) and the same cases using the Siemens LBM-simulated velocity fields from the Siemens CFD solver (b and d) under the same boundary conditions. Visual inspection ensured that main vortex core structures occurred in the same generalized location while Siemens CFD solver (b and d) resulted in a smoother core surface. Both cases had cores extracted using the same parameters: [normalized] $\overset{̿}{Q}$-criterion; threshold is their mean of [normalized] $\overset{̿}{Q}$, and only vortex cores with a volume > 0.5 mm^3^ were saved. Each vortex core was extracted from the cycle-averaged vorticity data (per respective case).

Vortex core results generated from the ANSYS-FLUENT and Siemens CFD solvers are summarized below. Only results from the [normalized] $\overset{̿}{Q}$-criterion method at a resolution of 0.2 mm were used. A visual comparison between the vortex cores generated from those two solvers was performed to ensure both their spatial position and quality. It was seen that both solvers generated results that led to the identification of the main vortex structure in a similar location. Two representative examples are provided in Figure 10. Visual inspection of the surface quality of identified vortex cores showed that cores from the Siemens LBM simulation had a smoother, less nebulous structure than that of the ANSYS-FLUENT simulation data.

**Figure 11:**
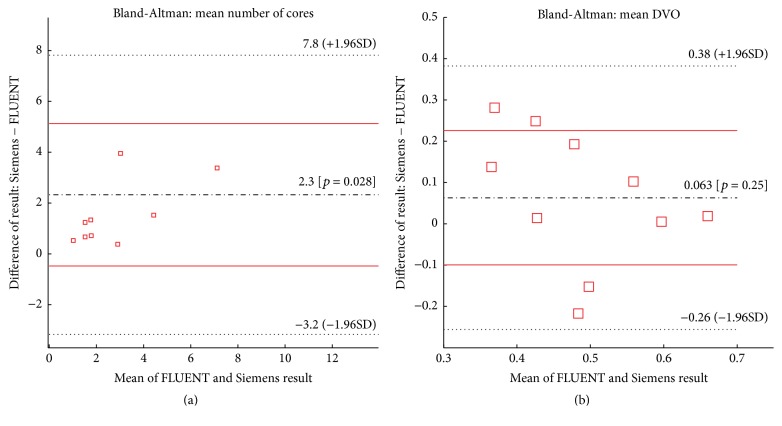
Bland-Altman plots showing the relation between two CFD platform results: (a) a mean number of cores and (b) mean DVO. The directionality of the BA plots is the Siemens CFD values minus the ANSYS-FLUENT CFD values.

Analyses of Bland-Altman method [33] were performed as a means to identify the difference between the two CFD platforms (Figure 11). In each plot of Figure 11, solid and dashed horizontal lines represent the limits of agreement (95% confidence interval) and the biases, respectively. Middle (black) lines in Figures 11(a) and 11(b) calculated by the Bland-Altman method indicate there were minimal biases found between the two above-mentioned CFD solvers in terms of the number of vortex cores and the DVO.

## 4. Discussions

Recently, using “image-based” CFD simulations to characterize intra-aneurysmal blood flow, with the primary goal of searching for correlations between local hemodynamics and the risk of aneurysm rupture, has drawn significant interests. In order to impact the clinical management of IAs, aneurysmal hemodynamics have to be processed in a clinical workflow. However, visual assessment of 4D hemodynamic characteristics, through pattern recognition using hundreds of cross-sectional images from multiple cardiac phases, may impose an extensive burden on reviewing physicians. For instance, spatiotemporal characteristics of vorticity patterns, which are the primary objective of this study, are difficult to assess through visual inspection of time-resolved 3D velocity vector fields. Having a computational methodology that can isolate and assess characteristics of vortex cores as a means to understand hemodynamic patterns within an IA may lead to valuable insight toward aneurysm risk assessment and is advantageous as compared to visual assessments of aneurysmal hemodynamics.

For this study, as inspired by an early work by Byrne et al. [16], we proposed an alternate method to identify vortex cores. In the original study by Byrne et al. [16], vortex core lines were extracted from tetrahedral meshes as discrete line segments. They used the length change of vortex core lines during a cardiac cycle as a surrogate for “flow complexity.” However, in the current study, vortex core patterns were derived based on isosurface extraction of the [normalized] $\overset{̿}{Q}$ values (see (3)). Overall, the initial results (Tables 1 and 2) showed that DVO had no significant correlation with several known geometrical parameters for individual aneurysm types (TA and SA), while the normalized variation (coefficient of variation) of the number of vortex cores only correlated with the aspect ratio of SA. This shows that the two vortex characteristic parameters do not lead to redundant information when compared to geometrical parameters. In addition, limited correlation could be found between the vortex core characteristics and the hemodynamic parameters of STA-WSS and SA-OSI. Therefore, our work introduced two additional parameters, which potentially contribute to a fuller feature space toward assessing swirling aneurismal flow patterns. More work is planned to further test the correlation between the proposed vortex parameters and other important hemodynamic parameters suggested by Chung and Cebral [39].

Our preliminary results (see Table 1) indicated that the mean DVO values were lower (not statistically significant, possibly due to the low number of cases) in SAs as compared to those in TAs. That is consistent with an anecdotal observation that (temporal) swirling flow patterns are more versatile among sidewall aneurysms over a cardiac cycle.

In Section 3.5, we compared results between two different solvers. The primary goal was to demonstrate that the proposed method can work well with velocity data in a rectilinear grid. Our initial visual inspection (Figure 10) showed that the placement and relative shape of vortex cores identified by both solvers were fairly consistent. While a bias toward an increased number of identified cores existed in the Siemens CFD solver over the ANSYS-FLUENT solver, the degree of difference remained minimal (Figure 11(a)). However, comparisons of DVO values showed larger discrepancies (Figure 11(b)) which were not surprising. Recall that vortex core region in this study was derived from the local velocity gradient matrices. Small differences in a 3D velocity field may be amplified during the process of calculations of *Q* and [normalized] $\overset{̿}{Q}$ values (see (3)). Nevertheless, these discrepancies require more careful investigations in future studies.

Although the proposed method could theoretically work with PC-MRI data, which are in rectilinear grids, more work is needed in order to explore whether or not the proposed method is directly applicable. This is because measurement noise in PC-MRI-measured velocity fields could be amplified when the velocity gradient matrices are being evaluated (see (2)). Furthermore, the spatial resolution of PC-MRI is still limited and is typically between 0.5 mm and 1.0 mm voxel size. The relatively large voxel size may compromise our ability to accurately extract clinically relevant vortex core regions (see Figure 7) as this method is susceptible to variations in voxel sizes. Additional studies will be needed to understand the proposed characteristics of the vortex, in which whether a broader view (larger voxel size) of the vortex structure is still able to help identify clinically relevant information toward aneurysm rupture risk.

Our results in Section 3 indicate that both the threshold values used for vortex identification and the resolution of velocity data impact the DVO and number of identified cores in an IA. This limitation is similar to what is known in image segmentation. For this feasibility study, in order to mitigate this shortcoming posed by threshold selection, we adopted the [normalized] $\overset{̿}{Q}$-criterion method for two reasons. First, we found that the adaptation of [normalized] $\overset{̿}{Q}$-criterion condensed the overall range of the *Q* or [normalized] $\overset{̿}{Q}$ values. This condensing of threshold values helps determine an acceptable range, while mitigating some of the large-scale vortex changes (less change in terms of the absolute volume change as shown in Figures 4(a) and 4(b)) that occur with the standard *Q*-criterion. Second, our preliminary results suggested that using the [normalized] $\overset{̿}{Q}$-criterion method helped identify the central structures of vortex patterns (see Figure 6), while limiting the identification of outlying weaker vortex patterns unless very low threshold values are used (Figure 5). Inclusion of weak boundaries of the vortex structure may indeed result in large, improper variations in estimated DVO values. In future studies, we intend to develop more automated methods based on machine learning [40]. The integrated machine learning approach will directly connect how the choices of vortex threshold value(s) and the resolution of velocity data impact the extraction of clinically relevant information. Nevertheless, the above-mentioned limitations have to be addressed through additional studies, particularly, through clinical studies, to determine clinical values of the proposed method of vortex core analysis.

Other limitations of our study include the small sample size and the use of standardized flow blood waveforms. However, our study design is still appropriate study design because our primary objective was to evaluate the proposed method of vortex core analysis using velocity data obtained from “patient-specific” CFD simulations. To keep this study concise, we limited the scope of the current study. 

## 5. Conclusions

A vortex core analysis algorithm for time-resolved 4D velocity fields was presented as a means to gain novel insight into aneurysmal hemodynamic characteristics. The proposed method was designed to extract vortex characteristics from velocity data in a rectilinear grid and therefore could be used for both CFD simulated data and, in theory, PC-MRI measured velocity field. Although this technique probably only represents an alternative to other plausible approaches, it does, in our opinion, represent a feasible path to make intra-aneurysmal hemodynamic assessments more quantitative, thereby enabling studies and comparisons of large populations both initially and over time. While this current method gives no additional direct insight toward risk of aneurysm rupture and is susceptible to variations in threshold values and resolution of velocity data, the preliminary results are encouraging in that our findings do not have a high degree of correlation with commonly used geometrical and hemodynamic variables. We believe that the method warrants further studies to explore its full clinical utility and significance.

## Figures and Tables

**Table 1:** A summary of three vortex core parameters in all 10 IA cases: average vortex volume, mean and standard deviation of the number of vortex cores across the cardiac cycle, and mean and standard deviation of DVO across the cardiac cycle. The data are presented as mean ± one STD.

Aneurysm	Mean vortex volume (mm^3^)	Mean number of cores	Mean DVO
TA1	2.015	1.05 ± 0.22	0.30 ± 0.08
TA2	8.83	3.67 ± 0.58	0.59 ± 0.15
TA3	2.02	1.19 ± 0.40	0.65 ± 0.15
TA4	3.04	2.71 ± 0.64	0.57 ± 0.12
TA5	0.50	0.76 ± 0.77	0.38 ± 0.39

SA1	0.84	1.09 ± 0.44	0.51 ± 0.19
SA2	4.78	2.90 ± 1.22	0.42 ± 0.18
SA3	0.69	0.90 ± 0.77	0.23 ± 0.22
SA4	2.57	1.43 ± 0.51	0.59 ± 0.14
SA5	55.46	5.43 ± 1.66	0.30 ± 0.068

**Table 2:** Pearson's linear correlation between geometrical parameters of IAs and characteristics of their vortex structures.

	Terminal aneurysm		Sidewall aneurysm		Combined data	
	Correlation coefficient	*p* value	Correlation coefficient	*p* value	Correlation coefficient	*p* value
*Mean number of vortex cores versus*						
Aneurysm volume	0.14	0.82	0.99	**<0.001**	0.81	**0.004**
Ostium area	0.15	0.81	0.82	0.086	0.62	0.055
Ostium circumference	0.12	0.84	0.77	0.12	0.56	0.092
Aneurysm height	0.73	0.16	0.91	**0.034**	0.83	**0.003**
Aspect ratio	0.60	0.29	0.66	0.23	0.62	0.058
Volume/ostium	0.23	0.71	0.99	**<0.001**	0.83	**0.003**
*Mean DVO versus*						
Aneurysm volume	−0.52	0.37	−0.42	0.49	−0.42	0.23
Ostium area	−0.43	0.47	−0.69	0.20	−0.63	**0.049**
Ostium circumference	−0.42	0.49	−0.74	0.15	−0.64	**0.046**
Aneurysm height	0.036	0.95	−0.20	0.75	−0.23	0.53
Aspect ratio	0.44	0.46	0.42	0.48	0.42	0.22
Volume/ostium	−0.64	0.25	−0.35	0.56	−0.37	0.29

**Table 3:** Tabulated results showing Pearson's linear correlation between the coefficient of variations of the number of cores and geometrical parameters of IAs.

Variation of vortex core number	Terminal aneurysm		Sidewall aneurysm		Combined data	
	Correlation coefficient	*p* value	Correlation coefficient	*p* value	Correlation coefficient	*p* value
Aneurysm volume	−0.54	0.34	−0.47	0.43	−0.20	0.58
Ostium area	−0.65	0.24	−0.06	0.92	−0.21	0.55
Ostium circumference	−0.68	0.21	0.038	0.95	−0.26	0.46
Aneurysm height	−063	0.26	−0.58	0.31	−0.36	0.31
Aspect ratio	0.28	0.65	−0.94	**0.016**	−0.26	0.46
Volume/ostium	−0.11	0.86	−0.53	0.36	−0.20	0.57

**Table 4:** Tabulated results showing Pearson's linear correlation between the coefficients of variations of the number of cores and mean DVO to the hemodynamic characteristics of IAs.

	Terminal aneurysm		Sidewall aneurysm		Combined data	
	Correlation coefficient	*p* value	Correlation coefficient	*p* value	Correlation coefficient	*p* value
*Coefficient of variation*						
STA-WSS	−0.69	0.20	0.90	**0.04**	−0.059	0.87
SA-OSI	−0.34	0.57	0.18	0.77	−0.017	0.96
*Mean DVO*						
STA-WSS	0.49	0.41	−0.18	0.77	0.11	0.75
SA-OSI	0.44	0.46	−0.30	0.63	−0.15	0.68

## Abbreviations

3D-DSA: Three-dimensional-Digital Subtraction Angiography
BA: Basilar artery
CFD: Computational fluid dynamics
DVO: Degree of vortex overlap
IA: Intracranial aneurysm
ICA: Internal carotid artery
LBM: Lattice-Boltzmann Method
MR: Magnetic resonance
PC-MRI: Phase-contrast magnetic resonance imaging
OSI: Oscillatory shear index
SA-OSI: Spatially Averaged Oscillatory Shear Index
STA-WSS: Spatially and Temporally Averaged Wall Shear Stress
STD: Standard deviation
STL: Stereolithography
TA-WSS: Temporally Averaged Wall Shear Stress
WSS: Wall shear stress
VTK: Visualization Toolkit
VMTK: Vascular Modeling Toolkit
3D: Three-dimensional.
